# Evaluation of the Pulmonary Veins and Left Atrial Volume using Multidetector Computed Tomography in Patients Undergoing Catheter Ablation for Atrial Fibrillation

**DOI:** 10.2174/157340309787048121

**Published:** 2009-01

**Authors:** Hiroki Ito, Khaled A. Dajani

**Affiliations:** Division of Cardiology, Loyola University Medical Center, Maywood IL-60153, USA

**Keywords:** Cardiac imaging, Multi-detector computed tomography, left atrial volume, pulmonary veins, atrial fibrillation, catheter ablation.

## Abstract

Catheter ablation is an evolving treatment option in patients with atrial fibrillation. Contrast enhanced electrocardiogram-gated multi-detector computed tomography (MDCT) has rapidly evolved over the past few years into an important tool in the diagnosis of coronary atherosclerosis. There is increasing recognition that MDCT is a useful tool to evaluate non-coronary structures, such as cardiac chambers, valves, the coronary sinus and adjacent structures including pulmonary veins. In particular, MDCT is playing an increasingly important role in the evaluation of the left atrium and the pulmonary veins in patients undergoing catheter ablation for atrial fibrillation. It provides accurate and reliable identification of the pulmonary veins and anatomical relationship between the left atrium and esophagus although the mobile esophagus may limit the value of MDCT to reduce the risk of atrio-esophagus fistula. In this article, we will review the evaluation of the left atrium and pulmonary veins using MDCT in patients undergoing catheter ablation of atrial fibrillation.

## CLINICAL BACKGROUND AND CATHETER ABLATION OF ATRIAL FIBRILLATION 

Atrial fibrillation (AF) is the most common sustained arrhythmia encountered in clinical practice. Its prevalence increases with age and in patients with hypertension or structural heart disease [1]. Atrial fibrosis is the likely structural substrate of AF [2]. In certain patients with risk factors, AF promotes thrombus formation most commonly in left atrial appendage, and requires chronic anticoagulation therapy [3]. In addition, AF is associated with poor exercise capacity, an increased risk of heart failure and all-cause mortality although its causality remains unclear [4,5]. Controversy surrounds the treatment strategies with AF: maintenance of sinus rhythm (rhythm control) versus rate-control [6-9]. A numbers of studies have failed to show a benefit with rhythm-control, and the AFFIRM trial suggested a trend toward lower mortality with rate-control [6-9]. However, these results may be explained by a high rate of AF recurrence in these trials and the thromboembolic events that occurred after discontinuing anticoagulation therapy in patients assigned to rhythm-control arm [6-9].

Since Haissaguerre M, *et al*. reported successful elimination of AF with targeted ablation of foci in or near the ostia of the pulmonary veins (PVs); catheter ablation of AF quickly became an established treatment option for symptomatic patients with AF refractory to antiarrhythmics [10,11]. A number of approaches have been developed, but they fall into two broad categories: PV isolation (PVI) and circumferential left atrial catheter ablation (LACA) [12,13]. There may be an increased rate of adverse effects with more extensive circumferential ablation. They have been reported to include left atrial flutter, pericardial effusions, and atrio-espphageal fistula [14,15]. Atrio-esophageal fistula is an often lethal complication, and has been associated with more extensive LACA procedures [14,15]. 

### Importance of the Pre-Procedural Imaging the Left Atrium and Pulmonary Veins in Catheter Ablation for Atrial Fibrillation

**Fig. (1):**
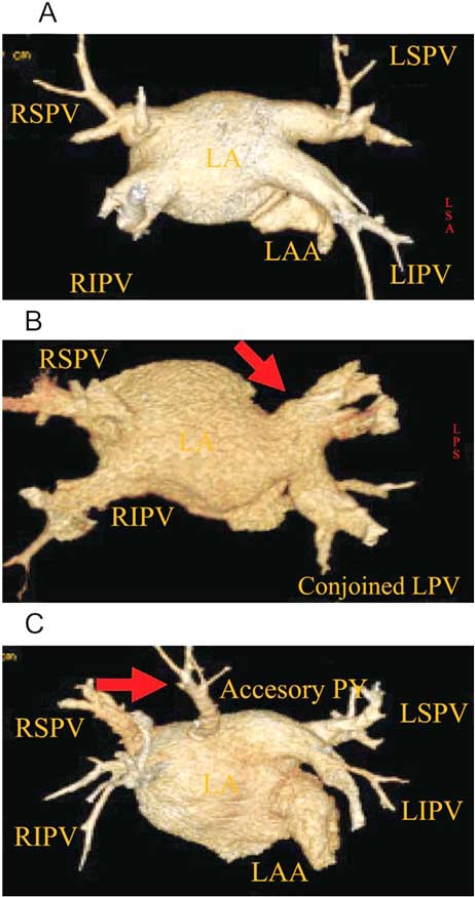
Variations in pulmonary vein patterns. A: Normal pulmonary veins; B: Conjoined left pulmonary veins; C: Accessory pulmonary vein.

Catheter ablation of AF commonly involves mapping and ablation of PV electrical activities near the venous ostia to electrically isolate the veins from the left atrium. For this approach, it is important to accurately assess the number and anatomy of the PV ostia so that all veins can be treated. In addition, the ostial dimensions are important in the selection of appropriately sized circular mapping catheters. The PVs exhibit a complex anatomy with significant inter-patient and intra-patient variability in size, shape, bifurcation, and branching pattern [16]. These variants include a common PV ostium (cojoined PV) and supernumerary (accessory) PV (Fig. **1**). The variability in PV anatomy makes AF ablation more challenging. Pre-procedural characterization of PV anatomy in a patient undergoing catheter ablation of AF facilitates selection of the ablation strategy and helps in guiding the ablation procedure in order to achieve an optimal result and minimize the risk of complications. 

### Comparison of Imaging Modalities to Visualize the Left Atrium and Pulmonary Veins

A variety of cardiac imaging modalities including conventional fluoroscopy/angiography, transesophageal echocardiography (TEE), intra-cardiac echocardiogram (ICE), multi-detector computed tomography (MDCT) and magnetic resonance imaging (MRI), have been used for catheter navigation during ablation procedures [16-18]. Pulmonary angiography with multiple wedge injections into the pulmonary arteries, and selective PV angiography after trans-septal puncture were considered to be the standard diagnostic techniques for identification of PV abnormalities but are invasive and may provide inaccurate measurement due to projection errors [17]. TEE is suboptimal for proper atrio-venous junction evaluation and is inadequate for visualization of more proximal portions of the PVs [17]. MDCT and MRI of the left atrium and PVs may be the most appropriate techniques with which to define the morphology and size of PVs before ablation procedures [19,20]. MRI is contraindicated in patients who have pacemakers or defibrillators, and its usage is limited by time-consuming acquisition processes. Although, to our knowledge, no studies have been published that compare the relative merits of CT versus MRI in this context, MDCT is becoming a more popular non-fluoroscopic navigation tool during catheter ablation for AF. 

Wood MA, *et al. *reported that MDCT identifies the greatest number of PV ostia among 4 modalities including MDCT, ICE, TEE, and venography, and its estimations of PV ostial dimensions are all significantly correlated with the dimensions estimated by ICE (r=0.57) and venography (r=0.52) [17]. PV ostial diameters were underestimated by TEE and overestimated by venography, compared with MDCT or ICE [17]. Furthermore, the study showed MDCT could detect more additional PV branches than ICE, and ICE underestimated PV ostial diameters [18].

Left atrial volume (LAV) has been reported to be a superior measure as a predictor of the cardiovascular outcomes in broad patient populations [21,22]. Transthoracic echocardiography remains the most widely used method for evaluation of left atrial size and function. Indeed, 2-dimensional (2-D) and 3-dimensional (3-D) echocardiography provide an accurate and reproducible estimate of LAV, and they allow us to evaluate PV inflow and trans-mitral inflow patterns [23,24]. Besides, TEE is considered the standard of reference for exclusion of atrial or atrial appendage thrombi [25]. However, echocardiography has some critical limitations. First, the quality of echocardiographic images can be limited by patient’s body such as obesity and obstructive lung disease. Second, the left atrium is not a symmetrically shaped 3-D structure, and left atrial enlargement may not occur in a uniform fashion. Therefore, one-dimensional assessment is unlikely to be a sensitive assessment of left atrial size change. Even 2-D echocardiographic assessment of left atrial volume may be inaccurate; it was reported to underestimate LAV systematically when compared with MDCT or MRI quantitation [19,20].

### Applications of MDCT in the Evaluation of Left Atrium and Pulmonary Veins in Patients Undergoing Catheter Ablation of Atrial Fibrillation

**Fig. (2):**
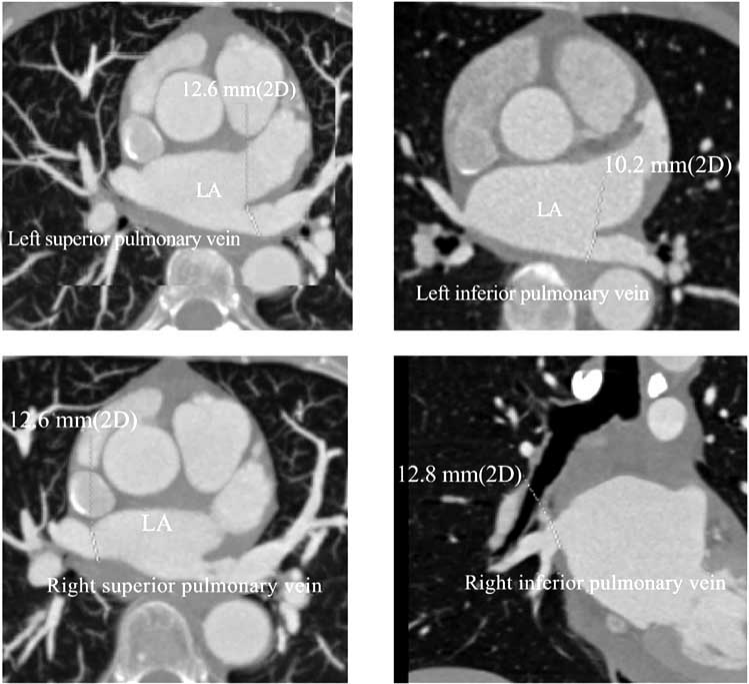
Measurements of size of pulmonary vein ostium.

Prospective electrocardiogram (ECG)-gating has been commonly used as a method to evaluate the left atrium and PVs [26]. Construction of 3-D left atrium and PVs model is processed using volume analysis software. Identification of the number and size of the PVs, draining pattern into the left atrium are assessed from the axial source slices. PV ostial diameters defined as the longest distance between two ostial points in the same plane in two orthogonal views are traced manually (Fig. **2**). The 3-D images of left atrium can be acquired using different acquisition techniques with MDCT. Prospective ECG-gating acquisition and construction of the images in diastole seem commonly used for LAV measurement [26]. However, the specific gating protocol was not disclosed in most of the previous reports, while there were only a few reports using the retrospective protocol [26-28]. To the best of our knowledge, no study comparing the merits of prospective versus retrospective gating in the evaluation of left atrial volume and function has been published to date. 

**Fig. (3):**
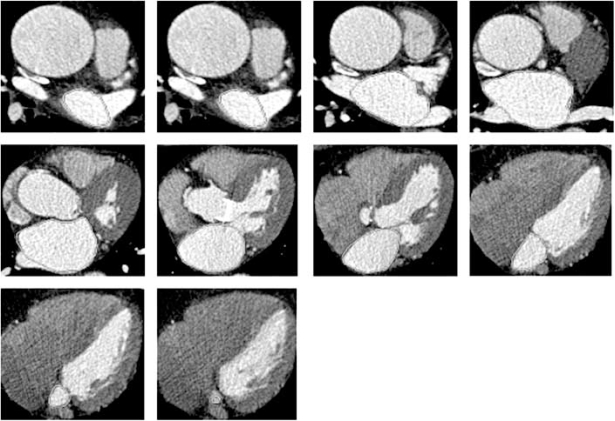
Paintbrush technique to calculate left atrial volume at each slice, excluding pulmonary veins and left atrial appendage.

**Fig. (4):**
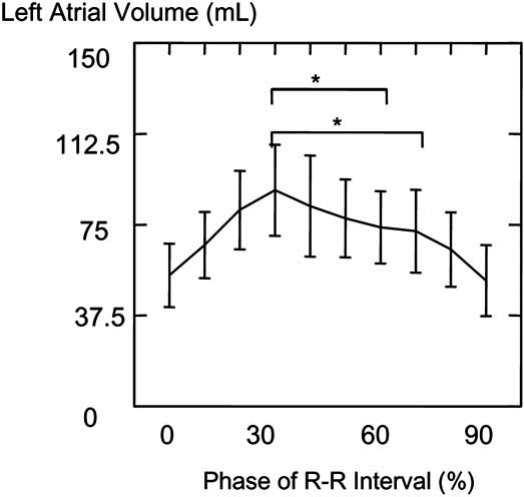
Left atrial volume (mean value ± standard deviation) in each phase of the R-R interval using retrospective ECG-gating method, *: p <0.001.

We conducted the study analyzing the LAV of 30 patients referred for cardiac MDCT using retrospective gating to see if one phase can accurately be used routinely for maximal LAV. LAV was measured by manually tracing the left atrium in each slice of the CT scan from the level of the mitral annulus to the roof of the left atrium. The left atrial appendage and PVs are excluded at their ostia (Fig. **3**). Of a total 30 patients, 17 patients (56.7%) had maximal LAV at 30% phase of the R-R interval, and 23 patients (76.7%) had minimal LAV at 90 % phase (Fig. **4**). Compared with retrospective gating method, prospective gating at 60% or 70% of the R-R interval significantly underestimated maximal LAV with a mean difference of 17.5±7.9mL and 17.5±8.8mL, respectively. Minimal LAV and LAEF could only be calculated using retrospective method (Table **1**).

Acquired PV stenosis after AF ablation is rare but serious complication of misplaced radiofrequency energy within the PVs [15]. The evaluation for PV stenosis is done by inspection of the PV in multiple projections for change in luminal diameter.

**Fig. (5):**
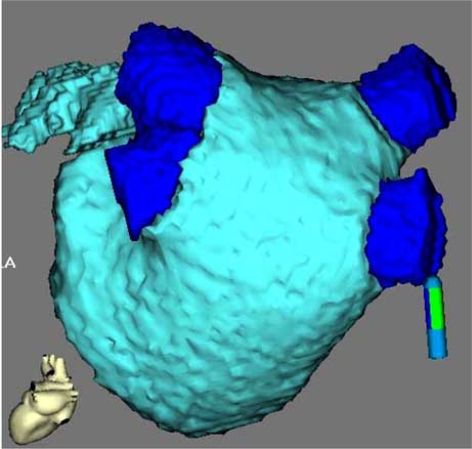
Left atrium and pulmonary veins: Integrated images of electro-anatomical mapping with three-dimensional computed tomographic images.

Electro-anatomical map (EAM) technology is the traditional method used to provide anatomic guidance for techniques used to isolate the PVs [29]. Creating an EAM is time consuming and may be technically difficult. MDCT images can be integrated with EAM data to allow the electrophysiologist to track ablation sites on a 3-D anatomical model of the left atrium and PVs. (Fig. **5**) [30,31]. The MDCT images integrated into an EAM or fluoroscopy were shown to reduce the fluoroscopic time and improve the success of catheter ablation of AF [30-32].

In addition, it was reported that the MDCT images could depict the anatomical relationship between the left atrium and esophagus [33,34]. On the other hand, Good, *et al.* showed that the esophagus was often mobile and shifted sideways by ≥2cm in a majority of patients undergoing catheter ablation under conscious sedation. Therefore, real-time imaging of the esophagus such as fluoroscopy may be necessary to reduce the risk of the thermal injury to the esophagus during the procedure [35].

There are several drawbacks for the routine use of MDCT for evaluation of the left atrium and PVs. Radiation dose is a concern especially in young women [36]. The typical radiation dose for a cardiac structure evaluation is on the order of 10mSv. This exposure can be reduced by ECG attenuation techniques that limit exposure during less informative parts of the cardiac cycle. Since iodinated contrast is used patients with decreased creatinine clearance should be hydrated to avoid contrast nephropathy. Gating remains a limiting factor in patients with fast and irregular heart rates. Finally obesity remains an issue despite attempts to optimize acquisition protocol. 

## CONCLUSION

Catheter ablation is an evolving treatment option in patients with AF. MDCT is playing an increasingly important role in the evaluation of the left atrium and PVs in such patients. It provides accurate and reliable identification of the PVs and anatomical relationship between the left atrium and esophagus although the mobile esophagus may limit the value of MDCT to reduce the risk of atrioesophagus fistula. Retrospective ECG-gating is necessary for accurate assessment of LAV and LAEF.

## Figures and Tables

**Table 1:** Comparison of Left Atrial Volume Variables Between 64 Slice CT with Retrospective ECG Gate and Prospective ECG Gate

	Retrospective ECG gate	Prospective gate at 60% RR interval	Prospective gate at 70 % RR interval	p Value
Phase of RR interval with max LAV (%) †	32.3±6.3	60	70	<0.001
Max LAV (ml)	93.9±32.9	76.4±28.1	75.0±30.4	<0.001
Mini LAV (ml)	51.5±30.2	NA	NA	NA
LA output (ml)	42.4±16.8	NA	NA	NA
LAEF (%)	46.8±16.5	NA	NA	NA

† : For calculation, phase of RR interval was considered continuous valuables.

ECG: electrocardiogram, LAV: left atrium volume, LAEF: left atrial ejection fraction, NA: not available
